# Potential impact and mechanism of Long Non-coding RNAs on cancer and associated T cells

**DOI:** 10.7150/jca.58859

**Published:** 2021-06-11

**Authors:** Wenxiu Chen, Shuna Liu, Fang Wang

**Affiliations:** 1 Department of Laboratory Medicine, the First Affiliated Hospital of Nanjing Medical University, Nanjing, China, 210029.; 2 National Key Clinical Department of Laboratory Medicine, Nanjing, China, 210029.

**Keywords:** Long non-coding RNAs, cancer, T cell

## Abstract

The discovery of many aberrant expressions of long non-coding RNAs (lncRNAs) in various cancers has focused attention on the effects of lncRNA on cancer cells themselves, including cell proliferation, growth inhibition, cell migration, cell immortality, vascular regeneration and cell viability. But with the increasing role of immunotherapy in cancer therapy, a large number of studies have revealed that the regulatory role of lncRNAs in immunity such as differentiation of immune cells can also influence the development and progression of cancer. In particular, recent publications have suggested that lncRNAs play critical roles in T-lymphocyte activation, proliferation, differentiation, function, apoptosis and metabolism. To elucidate the actual functions of lncRNAs at the molecular level of cancer pathogenesis, we summarize some of the current lncRNA regulatory mechanisms associated with T cell to discuss their effects in cancer in the hope of providing potential cancer therapeutic targets or cancer biomarkers. However, we all know that the differentiation and function of T cells is an extremely complex process that involves the expression and regulation of multiple lncRNAs. As a result, more regulatory mechanisms of lncRNAs need to be further studied.

## 1. Introduction

The regulatory role of non-coding RNAs (ncRNAs) in cancer has been favored by researchers in recent years. Among those, lncRNAs have gradually become an indispensable effective and regulatory component in different types of cancer, thus showing potential diagnostic and therapeutic value of cancer 1. The involvement of lncRNAs in cancer not only interacts with the cell cycle and proliferation pathways of cancer cells, but also effects the immune system by regulating tumor microenvironment (TME), epithelial-mesenchymal transition (EMT), microbiota, metabolism, immune cell differentiation and function of immune cells 2.

The proper functioning of the immune system is the key to fighting off pathogens and cancers. Meanwhile, lncRNAs have been found to be involved in the regulation of gene expression in the immune system, affecting the differentiation and function of various cell types of innate immunity and adaptive immunity such as macrophage myeloid cell and T cell 3. T cells, the most important participants in the process of innate and adaptive immunity, play a crucial role in regulating the immune response. The normal proliferation, differentiation and function of T cells is critical to maintaining the appropriate execution of the immune system. Many studies underscore the important role of lncRNAs in CD4^+^ T cell differentiation and have shown that the abnormal differentiation of CD4^+^ T-helper (Th) cells is influenced by lncRNAs and may lead to autoimmune diseases 4, 5. Advances in analytical methods like real-time fluorescent quantitative PCR (qPCR) and microarrays have enabled more and more people to analyze the differential expression of lncRNAs in different T cell subtypes and at different stages of T cell growth and development. For example, Jinping Zhang et al. examined the expression profiles of lncRNAs and mRNAs in CD4^-^CD8^-^ (DN), CD4^+^CD8^+^ (DP), CD4^+^CD8^-^ and activated CD4^+^CD8^-^ T cells in a microarray analysis and found specific expression profiles of lncRNAs at different stages of development 6. Gangqing Hu et al. performed RNA-Seq of peripheral T cells at multiple time points during their differentiation and confirmed that the expression of lncRNA is cell-specific 7. More importantly, one research found that immune-related lncRNAs in lung cancer patients have significantly increased expression in T cells 8. Thus, in this review, we will provide an overview of current state of knowledge implicating lncRNAs and T cells, with main emphasis on the regulatory effects of lncRNAs on the activation, proliferation, differentiation, effector functions, apoptosis and metabolism of T cells and their potential implications in cancer.

## 2. Long non-coding RNAs

It is now well known that the human genome is almost completely transcribed 9. However, numerous studies have shown that only a small fraction of the transcriptome is translated, and that most transcriptional output is ncRNA. LncRNAs are non-coding RNAs with lengths greater than 200 nucleotides (nt) and do not have an open reading frame 10, 11. In this chapter, we will provide an overview of lncRNA biogenesis and function.

### 2.1. LncRNA biogenesis

LncRNA transcripts are longer than 200nt in length and they don't have the ability to code for proteins. Most known lncRNAs have been found to be transcribed by RNA polymerase II (Pol II). Therefore, they are structurally similar to messenger RNA (mRNA) and may have cap structures and poly A tails. The main difference between lncRNA and mRNA is that lncRNA contains transcriptional termination codons, so lncRNA has no protein-coding ability 12-16. However, recent ribo-seq and mass spectrometry data suggested that lncRNAs may be translatable due to their small open reading frames encoding for short peptides 17, 18. Liman Niu et al. even have verified a micropeptide encoded by lncRNA MIR155HG 19. LncRNAs are often classified into five groups based on their genomic positions relative to protein-coding genes: sense lncRNAs, antisense lncRNAs, intronic lncRNAs, bidirectional lncRNAs and intergenic lncRNAs. Overlap with one or more exons of the encoding gene are sense lncRNAs, antisense lncRNAs are complementary to the transcriptional products on the opposite chain, intronic lncRNAs are produced in the introns of genes, bidirectional lncRNAs and protein-coding genes share the same promoter but the transcriptional direction is reversed and intergenic lncRNAs are transcribed independently by sequences located between protein-coding genes 20-22.

### 2.2. LncRNA function

More and more studies have proposed the regulation mechanism of lncRNA in diseases. Studies have shown that lncRNA mainly regulates gene expression from three aspects: epigenetics, transcriptional regulation and post-transcriptional regulation 23-25, and more specifically, lncRNA regulates gene expression, chromatin organization, cellular trafficking, RNA decay and translation. Furthermore, it is worth noting that lncRNA will also affect protein localization, function, decay and turnover 26. LncRNA can play functions through RNA-DNA, RNA-RNA and RNA-protein interactions 27. Previous studies have shown mechanisms of action of lncRNA: (I) interfere with the expression of adjacent protein-coding genes by transcription in the upstream promoter region of protein-coding genes; (II) affect gene expression by inhibiting RNA polymerase, or by mediating chromatin remodeling and histone modification; (III) Interfere with the splicing of mRNA by supplementing the double chain with transcripts of protein-coding genes; (IV) use the transcripts of protein-coding genes to complement the double chains and regulate gene expression through the production of endogenous small interference RNA by dicer enzymes; (V) regulate activity by binding to specific proteins; (VI) form nucleic acid protein complexes with protein as their structural components; (VII) change the cytoplasmic localization of proteins by binding to specific proteins; (VIII) as precursor molecules of small RNAs 15.

## 3. LncRNAs and regulation of T cell activation, proliferation, differentiation, effector functions, apoptosis and metabolism

A better understanding of the role of lncRNAs in T cells with extensively investigated disease such as autoimmune disease may provide new lncRNA-T cell-based therapeutic target for cancer. Many lncRNAs have been explored for their role in regulating T cell activation, proliferation, differentiation, effector functions, apoptosis and metabolism. In this chapter, we will show some typical lncRNAs with fully-elucidated role in regulating T cell.

### 3.1. Activation

The activation of T cells depends on the double signal of antigen and costimulatory molecules. Antigen-presenting cell (APC) is a kind of immune cell that captures, processes and presents antigens to antigen-specific lymphocytes. APC provides at least two independent signals to stimulate T cell activation. Major histocompatibility complexes (MHC) and costimulatory molecules expressed by APC are the sites where many lncRNAs regulate. Dendritic cells (DC), the most important APC, has been found in many studies to be extensively regulated by lncRNA in the activation of T cells. Yiling Ding et al. have identified a lncRNA specifically expressed in DC can promote the over-maturation of DC and induce a strong immune response 28. On the contrary, many researchers have noticed that some lncRNAs can induce DC to become tolerogenic dendritic cells (tDC). For example, Jian Wu et al. demonstrated that lncRNA MALAT1 induces DC towards tolerant type and MALAT1 promoted dendritic cell-specific intercellular adhesion molecule-3 grabbing nonintegrin (DC-SIGN) expression by functioning as an miR155 sponge. This maintains tolerance of DC by secreting interleukin-10 (IL-10), leading to low level of CD80, CD86 and MHC expressed by DC 29. In addition to MALAT1, NEAT1 has been demonstrated to induce DC to develop into tolerogenic phenotype by regulating the NACHT, LRR, and PYD domains-containing protein3 (NLRP3) inflammasome as a competitor for miR-3076-3p 30.

LncRNA not only affects the double signal provided by APC cells for T cell activation, but also specifically affects the expression of MHC molecules and costimulatory molecules. Previous studies showed that lncRNA IFNG-AS1 is related to the production of IFN-γ, but in a study by Mengchuan Luo et al., they found IFNG-AS1 affects the expression of HLA-DRB1 to control the immune response in myasthenia gravis (MG) 31. Additionally, according to Liu Qian et al's research, lncRNA GAS5 upregulates adenovirus E4 binding protein 4 (E4BP4) which targets CD40L to inhibit activation of CD4^+^ T cell by inhibiting miR-92a-3p in systemic lupus erythematosus (SLE) 32. In addition to positive costimulatory molecules, negative costimulatory molecules represented by PD-1/PD-L1 have also attracted extensive attention in recent years. Multiple lncRNAs have been shown to be involved in the process. For instance, Bo Hu et al. revealed that lncRNA XLOC_003810 decreased the proportion of CD4^+^ PD-1^+^ T cells in patients with MG 33.

### 3.2. Proliferation and differentiation

Naive T cells are activated by double signals, proliferate and differentiate into different functional subsets under the action of microenvironmental cytokines. It has been well established that lncRNAs are also widely involved in the differentiation of helper T cells (Th) and regulatory T cells (Treg). For example, Martina Gast et al. used NEAT1^-/-^ mice as a model of lncRNA NEAT1 deficiency to show anomalous Treg and Th cell differentiation. They finally found a shift of CD4^+^ T cell balance towards Th cell proliferation both in the spleens and in the circulating blood 34. Furthermore, the differentiation of Th is also orchestrated by numerous lncRNAs. Many studies have shown that the differentiation of CD4^+^ T cells into Th17 cells is a major cause of the occurrence and development of autoimmune diseases. Xiaolong Shui et al. determined the involvement of lncRNA NEAT1 in the differentiation of Th17 cells by targeting STAT3 protein level in rheumatoid arthritis (RA) 35. Fang Zhang et al. determined lncDDIT4 inhibits DDIT4/mTOR signaling by targeting DNA-damage-inducible transcript 4 (DDIT4) to suppress Th17 cells differentiation in multiple sclerosis (MS) 36. RORγt is the main transcription factor for Th17 cell differentiation, and Manuel B. Braga-Neto et al. described a novel lincRNA XLOC_000261 which appears to negatively regulate RORγt protein expression in Th17 cells in Crohn's disease (CD) 37. Besides, Th1/Th2 lineage differentiation requires the involvement of many lncRNAs. Valeria Ranzani et al. found that down-regulation of linc-MAF-4 by mediating transcription of MAF through epigenetics enables T cells to differentiate toward the Th2 subtype instead of Th1 subtype 38. The signal transducer and activator of transcription (STAT) 6 is an important transcription factor for the differentiation of Th2 cells and Shuman Huang et al. demonstrated that lncRNA NEAT1 promotes Th2 differentiation through EZH2/ITCH/STAT6 axis 39. GATA3 is also considered the master regulator of Th2 and Hunter R. Gibbons et al. demonstrated that divergent lncRNA GATA3-AS1 is necessary for the transcription of GATA3, thus promoting the differentiation of Th2, but it cannot induce total Th2 polarization independent of other factors such as c-Maf or Stat6 40. Moreover, lncRNAs in exosomes have also been found to regulate T cell differentiation. Xiaoyuan Zhu et al. detected the expression of lncRNA GAS5 in exosomes which were isolated from allergic rhinitis (AR) patients and found GAS5 suppresses Th1 differentiation and promotes Th2 differentiation via downregulating EZH2 and T-bet 41. Similarly, lncRNAs play vital role in Treg differentiation. TGF-β signaling is an important pathway to induce Treg cells, and this process has also been shown to be actively regulated by lncRNA. For instance, Meng Xia et al. identified lnc-smad3 inhibits the H3K4 methyltransferase Ash1l-mediated Smad2/3-TGF-β signaling pathway and thus inhibits induced Tregs (iTreg) polarization 42. Treg-specific transcription factor Foxp3 plays a key role in the differentiation of Treg cells and it has been well documented that lncRNA regulates the expression of Foxp3 to promote Treg cell differentiation. Aleksandra Brajic et al. identified a novel lncRNA Flatr which anticipates Foxp3 expression in Tregs 43 and David Zemmour et al. found that lncRNA Flicr modulates Foxp3 expression through modification of chromatin accessibility 44.

### 3.3. Effector functions

Distinct effector T cell subsets have their own characteristics and effects. CD4^+^ T cells are divided into a series of multifunctional immune cell subsets, including Th1, Th2, Th17, and Treg cells as well as unconventional T helper subsets. Cytotoxic CD8^+^ T cells are the main component that clears virus infection and resists tumor in adaptive immunity. Different subtypes of Th cells secrete different cytokines to function, and the possibility that lncRNAs participate in this process has been investigated. IFN-γ production is considered to be the most representative marker of Th1 cells and is often associated with inflammation and autoimmune diseases. Studies have shown that lncRNA IFNG-AS1, also known as Tmevpg1, regulates the production of IFN-γ 45. For example, Carl Robert Rankin et al. verified IFNG-AS1 promotes the production of IFN-γ, thus exacerbating inflammatory bowel disease (IBD) 46 and Juan Wang et al. found TMEVPG1 enhances the immune response of Th1 cells in patients with Sjogren syndrome 47. In addition, a large number of other studies have shown that IFNG-AS1 enhances the immune response of Th1 and is associated with autoimmune diseases such as Multiple sclerosis (MS) and Hashimoto's Thyroiditis (HT) 48, 49.

Compared with Th1 cells, Th2 cells mainly mediate humoral immunity by secreting cytokines such as IL-4, IL-5, IL-6 and IL-10. Weijie Yin et al. have identified a noncoding interleukin 4 (IL-4) RNA that promotes IL-4 mRNA translation in Th2 50. James P. Hewitson et al. demonstrated lncRNA Malat1 suppresses immunity to infection through promoting expression of Maf, a key transcriptional regulator of IL-10 in Th cells 51. However, this finding is contrary to the conclusion of another study conducted by Yingpeng Yao and his colleagues that Malat1 is not essential for response to lymphocytic choriomeningitis virus (LCMV) infection 52. Of course, they used different models of mouse infection but actually further research has yet to be confirmed.

Th17 cells mainly secrete IL-17 to participate in the inflammatory response. Yuying Qiu et al. demonstrated lncRNA-MEG3 acts as a microRNA-17 sponge to promote the production of Th17-related cytokines such as IL-17 and IL-22 and disrupt the balance of Th17 and Treg cells, thus contributing to the pathogenesis of asthma 53. Likewise, Jianqin Li et al. also found that MEG3 induces the imbalance of Th17 and Treg cells by inhibiting the expression of miR-125A-5P in immune thrombocytopenic purpura (ITP) 54.

Treg cells are a group of T cells with immunosuppressive function, mainly including natural Tregs (nTregs) and induced Tregs (iTregs). Treg cells can reduce tissue damage caused by autoimmunity in chronic immune responses. In parallel, they also create opportunities for tumor immune escape. Many findings reveal the mechanisms by which Treg cells inhibit immune response. Xinhong Pei et al. demonstrated that lncRNA SNHG1 regulated IDO by targeting miR-448 to promote Treg cell differentiation and IL-10 secretion to mediate the immune escape of breast cancer (BC) 55. Jing Wang et al. demonstrated that lncRNA DQ786243 upregulates Foxp3^+^ Treg cells in oral lichen planus (OLP) and suppresses the secretion of IFN-γ and IL-17 by other CD4^+^ T cells such as Th1 and Th17 through Foxp3-miR-146a-NF-κB axis 56. In comparison to αβT cells, γδT cells have been less studied. But recently Chao Ni et al. showed that breast tumour cell-derived exosomes (TDEs) could transmit lncRNA SNHG16, which induces CD73^+^ γδ1 Treg cells through TGF-β1/lncRNA SNHG16/miR-16-5p/SMAD5 pathway. Notably, CD73^+^ γδ1 Treg cells inhibit CD4^+^ T cells from producing IFN-γ and CD8^+^ T cells from producing perforin and granzyme, and secreting IL-10 and TNF-β depending on the adenosine pathway 57.

Cytotoxic CD8^+^ T cell whose effector function is also regulated by lncRNA plays an irreplaceable role in the body's resistance to pathogen infection and tumor cells by secreting IFN-γ and TNF-α. Yang Wang et al. showed that lncRNA-CD244 which is induced by CD244^+^ CD8^+^ T cell in active tuberculosis (TB) infection inhibits IFN-γ/TNF-α expression in CD8^+^ T cells through recruitment of EZH2 to IFNG and TNFA loci for repressive chromatin states 58. Moreover, Jiansong Wu et al. have found that lncRNA‑CD160 bind to histone‑modification enzyme gene histone deacetylases 11 (HDAC11) to form a complex and inhibit the function of HDAC11, which further inhibits the secretion of IFN‑γ and TNF-α in in hepatitis B virus infection 59.

### 3.4. Apoptosis

LncRNA regulates extrinsic and intrinsic apoptosis of T cells. Extrinsic apoptotic pathway is mediated by death receptors such as tumor necrosis factor related apoptosis-inducing ligand (TRAIL) and Fas-ligand (Fas-L). Intrinsic apoptotic pathway refers to increased permeability of mitochondrial outer membrane. Di Huang et al. examined that the JAK1-STAT1 signaling pathway in activated T cells upregulates the expression of NKILA lncRNA and inhibits NF-κB activity, thus leading to sensitivity of tumor-specific cytotoxic T lymphocytes (CTLs) and type 1 helper T (Th1) cells to activation-induced cell death (AICD) in the tumor microenvironment of breast cancer and lung cancer 60. Similarly, a research found lncRNA TANCR regulates the cytotoxic function of γδ T cells by affecting the expression of TRAIL 61.

In addition, lncRNAs are widely involved in intrinsic apoptosis pathway. S. M. Ali Hosseini Rad et al. showed that a divergently transcribed lncRNA LOC107985203 negatively modulated Mcl-1 expression, a member of the Bcl-2 anti-apoptotic protein family to affect the lifespan of T lymphocytes 62. Besides, Wiam Saadi et al. have found a lncRNA XLOC_000895 (Robnr), located downstream of the anti-apoptotic gene Bcl2, affected the activation of Bcl2 in the development of T lymphocytes 63. In addition to influencing Bcl2 anti-apoptotic protein family, lncRNA was also found to be involved in the regulation of apoptotic factors such as Par-4. Lin Zhang et al. demonstrated that knockdown of a novel lncRNA, which is characterized from the Jurkat leukemic T-cell line can induce apoptosis of T-cell acute lymphoblastic leukemia (T-ALL) cells by promoting the formation of par-4 /THAP1 protein complex, thus increasing the activation of caspase-3 and the expression of pro-apoptotic smac protein 64. Apoptosis-inducing factor, mitochondrion-associated 2 (AIFM2) has also been found to be upregulated by lncRNA MEG3 through targeting miR-214 in T-cell lymphoblastic lymphoma (T-LBL) 65 Actually, considerable studies have explored specific lncRNAs involved in the regulation of the apoptosis of T lymphocytes, but the specific mechanism of apoptosis remains unclear. For example, Hongbing Liu et al. found that knockout of lncRNA NEAT1 increases sensitivity of Jurkat CD4^+^ T cell lines to apoptosis during HIV-1 replication, but no specific mechanism has been found 66.

### 3.5. Metabolism

The transition from naive T cells to activated T cells mediated by TCR and co-stimulation signals involves a metabolic transition from oxidative phosphorylation (OXPHOS) to glycolysis 67 although some studies have shown that Tregs and memory CD8^+^ T cells depend on OXPHOS 68. Many signaling pathways of TCR in the activation of T cells are associated with the metabolic process. Among those, PI3K/Akt/mTOR signaling pathway is demonstrated to affect transcription of several glycolytic genes 69. Jiayao Fu et al. have found that lncRNA PVT1 regulates the expression of Myc through the PI3K/Akt signaling pathway, thus affecting the activation and proliferation of CD4^+^ T cells by reprogramming glycolysis 70. Specific metabolic programs regulated by lncRNAs are adopted when T cells run through the activation, proliferation, differentiation and effector functions. LncRNA regulatory mechanisms responsible for key metabolic enzymes or transporters of energetic nutrients in the process may provide an explanation for metabolic reprogramming.

## 4. Role of lncRNAs in cancer associated with T cell

Considering the role of T cell in immunotherapy of cancer, lncRNAs involved in T cell activation, proliferation, differentiation, effector function, apoptosis and metabolism can impact cancer progression. Thus, lncRNAs can play an oncogenic or a cancer suppressive role through regulation of T cells, helping explore immunotherapy targets and overcome immunotherapy resistance in cancer. In this chapter, T cell associated lncRNAs in cancer and the possible pathways involved as well as potential clinic applications will be discussed.

### 4.1. T cell-associated lncRNAs in cancer

One of the central roles of T cell in immunity is the recognition and elimination of malignant transformations. Tumor cells inhibit T cell activation by reducing their antigenicity and upregulating immunosuppressive molecules. Qingsong Hu et al. reported that the transformed mammary gland epithelial cells downregulate antigen presentation machinery upon expression of LINK-A which facilitated crosstalk between phosphatidylinositol-(3,4,5)-trisphosphate and inhibitory G-protein-coupled receptor (GPCR) pathways, damping protein kinase A-mediated phosphorylation of the E3 ubiquitin ligase TRIM71, enhancing K48-polyubiquitination-mediated degradation of the antigen peptide-loading complex (PLC) in human triple-negative breast cancer (TNBC) 71 and Hao Li et al. demonstrated that long intergenic non-protein coding RNA 2195 (LINC02195) was closely related to antigen processing and presentation by affecting genes encoding MHC I molecules in head and neck squamous cell carcinoma (HNSCC) 72. Besides, the possibility that lncRNAs are involved in regulating the expression of PD1/PD-L1 in cancer and affecting the activation of T cells has been raised. For example, PD-L1 is upregulated in pancreatic cancer (PC) by lncRNA LINC00473 as a sponge of microRNA-195-5p 73 and lncRNA HOTTIP promotes IL-6 transcription by regulating transcription factor c-jun to activate the STAT3/PD-L1 pathway in neutrophils in ovarian cancer (OC) 74. Moreover, Hasmeena Kathuria et al. have found that genes regulated by NKX2-1-AS1 are related to PD-L1/PD-1 checkpoint pathways in human lung carcinoma 75 and Qing-Ming Wang et al. revealed that MALAT1, PD-L1 were upregulated in diffuse large B cell lymphoma (DLBCL) tissues by targeting miR-195 76. Compared with PD-L1, PD1 expression on lymphocytes was also found to be regulated by lncRNAs. For instance, AFAP1-AS1 and PD-1 are co-expressed in infiltrating lymphocytes in Nasopharyngeal carcinoma (NPC) 77.

Another central role of T cell immunity is the differentiation of distinct effector T cell subsets. Lnc-SGK1 induced by Helicobacter pylori infection and high salt diet suggests a possibility of promoting Th2 and Th17 differentiation in human gastric cancer (GC) by SGK1/Jun B signaling 78 and lnc-sox5 was found to promote the expression of indoleamine 2,3-dioxygenase 1 (IDO1) to modulate the infiltration and cytotoxicity of CD3^+^CD8^+^ T cells in colorectal cancer (CRC) 79. Additionally, differentiation of Tregs is associated with the progression of cancer. For instance, linc-POU3F3 activates TGF- β signal pathway to increase Treg distribution and thereby promote gastric cancer (GC) cell proliferation 80 and the transcription factor FOXC1 mediates LINC00301 increases Treg through targeting TGF-β in non-small cell lung cancer (NSCLC) is verified by Cheng-Cao Sun et al. 81.

**Figure 1:**
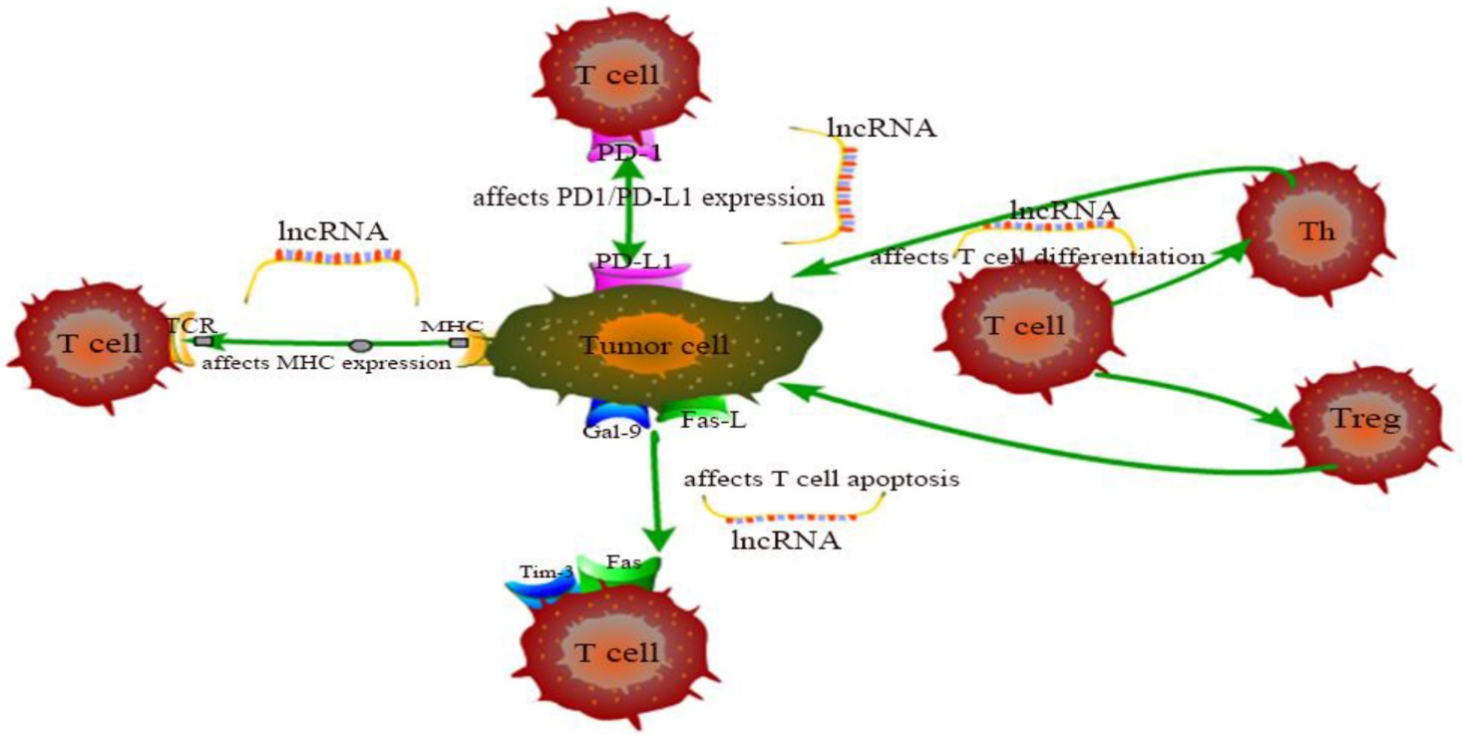
The specific regulation of lncRNAs on T cells.

The exhaustion and apoptosis of T cells affect central role of T cell immunity, and more and more studies have proved that lncRNAs are involved in the regulation of this process. For example, T cell immunoglobulin mucin 3 (Tim-3), an inhibitory receptor, is regulated by lncRNA to alter the immune response of CD8^+^ T cells. Jie Ji et al. demonstrated that lnc-Tim3 promotes T cell exhaustion via suppressing Tim-3-Bat3 signaling and downstream signaling pathway NFAT1 and AP-1 in hepatocellular carcinoma (HCC) 82. Similarly, Kai Yan et al. showed that down-regulation of lncRNA NEAT1 can limit the apoptosis of CD8^+^ T cells through miR-155/Tim-3 pathway in HCC 83. In the tumor microenvironment of breast cancer and lung cancer, Di Huang et al. examined that the JAK1-STAT1 signaling pathway in activated T cells upregulates the expression of NKILA lncRNA and inhibits NF-κB activity, thus leading to sensitivity of tumor-specific cytotoxic T lymphocytes (CTLs) and type 1 helper T (Th1) cells to activation-induced cell death (AICD) 60. Aside from that, Lalit Sehgal et al. found that a lncRNA corresponding to an antisense transcript of Fas (FAS-AS1) regulates alternative splicing of Fas in lymphomas and therefore impairing Fas-mediated apoptosis in B-cell lymphoma 84. These are just a few of lncRNA examples representative to carcinogenesis. LncRNAs involved in cancer regulation associated with T cells in this chapter are shown in Table 1 and the specific regulation of lncRNAs on T cells is shown in Figure 1. Although the regulation of lncRNAs in the pathogenesis of cancer is not fully understood, lncRNA-mediated T cell is an important direction.

### 4.2. Possible pathways of lncRNAs on cancer and T cell

LncRNAs are emerging as key regulators of gene expression and play a key role in the immunity related to progression of cancer. However, only a small number of T cell-associated lncRNAs as well as possible pathways have been found to play a role in cancer regulation. Yongsheng Li et al. demonstrated that not all immune-related pathways are equally associated with lncRNAs; cytokine and cytokine receptor pathways are likely to be correlated with more lncRNAs 8. In fact, chemokines and chemokine receptors have long been reported to become new targets for cancer immunotherapy 85 and Mu Xu et al. found that SATB2-AS1 inhibits colorectal cancer (CRC) cell metastasis and regulates TH1-type chemokines expression 86. Moreover, in the research conducted by Yongsheng Li et al., several immune-related lncRNA are associated with TCR signaling pathway, hippo pathway and cell cycle pathway in lung cancer 8. Based on this, we hypothesized that these signaling pathways are might also associated with T cells in cancer. Identification of the targets of lncRNAs remains to be further investigated.

### 4.3. Potential clinic applications of T cell-associated lncRNAs

Cancer-associated lncRNAs are being favored by researchers and widely studied. Adam M. Schmitt et al. summarized the mechanisms of cancer-related lncRNAs in the diagnosis, treatment and prognosis of various types of cancer 87. However, with the key role of immunotherapy in cancer, some researchers have not only focused on the regulation of lncRNAs on cancer cells, but also tried to find immune-related lncRNAs to explore new targets in cancer, and simultaneously make contributions to overcoming the resistance of immunotherapy. Yuwen Zhou et al. discussed the mechanisms of lncRNA participation in immunotherapy resistance from the aspects of antigen presentation, PD-L1 expression regulation, modulation of CD8^+^ T cells and control of Tregs and myeloid-derived suppressor cells (MDSCs) 88. In our review, we discussed the mechanisms by which lncRNAs affect T cell activation, differentiation, effector function and apoptosis in cancer. Upregulation or downregulation of related lncRNAs are also expected to help overcome immunotherapy resistance.

## 5. Discussion

The activation, proliferation, differentiation, effector functions, apoptosis and metabolism of T cell have been studied a long time ago and the regulation of lncRNAs in these processes has also attracted attention over the years. Although lncRNAs do not seem to be as critical as T cells themselves in cancer immunotherapy to some extent, it is obvious that lncRNAs are vital to this regulatory network by providing a background support to enable T cells to play a more effective role in cancer immunity.

The most important characteristic of lncRNA-mediated regulation on T cells is that lncRNAs can bind to signal molecules such as immune receptors and transcriptional regulators. Given the complexity of T cell differentiation and function, it is very difficult to try to elucidate carcinogenesis with the function of a single lncRNA. In the above review of lncRNAs in T cells, we found that in every disease model, multiple lncRNAs are involved in a complex regulatory network and the same lncRNA also plays different roles in different disease models. More importantly, some newly discovered lncRNAs have no clear molecular target and no signaling pathway has been found, leaving many difficulties for lncRNAs to be used as cancer therapeutic targets or cancer biomarkers in the future. The uncertainty of molecular targets also explains the functional diversity of lncRNAs. It is important to keep in mind that lncRNAs alone may not be sufficient to affect cancer immunity; likewise, T cells may not take effect on their own or they alone may not work efficiently in cancer immunity. Since the regulation of lncRNAs on T cells is still in the early stage of research, we hope to find more lncRNAs in regulation of T cells and their specific signaling pathways in the future. Further characterization of these lncRNAs will provide new insight into how lncRNAs and signaling molecules work together to impact T cell in cancer.

## Figures and Tables

**Table 1:** Summary of some representative lncRNAs involved in cancer regulation.

LncRNA	Function	Pathway	Cancer	Ref
LINK-A	Downregulates antigen presentation	GPCR	TNBC	71
LINC02195	Promotes MHC I molecules	Unknown	HNSCC	72
LINC00473	Upregulates PD-L1	miR-195-5p	PC	73
HOTTIP	Upregulates PD-L1	STAT3/PD-L1	OC	74
NKX2-1-AS1	Downregulates PD-L1	NKX2-1/CD274	LC	75
MALAT1	Upregulates PD-L1	miR-195	DLBCL	76
AFAP1-AS1	Upregulates PD-1	Unknown	NPC	77
Lnc-SGK1	Promotes Th2 and Th17 differentiation	SGK1/Jun B	GC	78
Lnc-sox5	Downregulates CD3^+^CD8^+^ T cells	IDO1	CRC	79
Linc-POU3F3	Increases Treg distribution	TGF-β	GC	80
LINC00301	Increases Treg distribution	TGF-β	NSCLC	81
Lnc-Tim3	Promotes CD8^+^ T cell exhaustion	Bat3/NFAT1/AP	HCC	82
NEAT1	Promotes the apoptosis of CD8^+^ T cells	miR-155/Tim-3	HCC	83
NKILA	Sensitizes CTL, Th1 to AICD	JAK1-STAT1	BC, LC	60
FAS-AS1	Upregulates Fas	Unknown	BCL	84

TNBC: triple-negative breast cancer; HNSCC: head and neck squamous cell carcinoma; PC: pancreatic cancer; OC: ovarian cancer; LC: lung cancer; DCBLC: diffuse large B cell lymphoma; NPC: nasopharyngeal carcinoma; GC: gastric cancer; CRC: colorectal cancer; NSCLC: non-small cell lung cancer; HCC: hepatocellular carcinoma; BC: breast cancer; BCL: B-cell lymphoma; GPCR: G-protein-coupled receptor.
